# Efficacy and safety of Glofitamab in patients with R/R DLBCL in real life setting– a retrospective study

**DOI:** 10.1007/s00277-025-06438-3

**Published:** 2025-06-13

**Authors:** Ronit Gurion, Dmitri Guz, Meirav Kedmi, Chava Perry, Irit Avivi, Netanel A. Horowitz, Uri Abadi, Anat Gafter‑Gvili, Pia Raanani, Neta Goldschmidt, Boaz Nachmias, Shlomzion Aumann

**Affiliations:** 1 Institute of Hematology, Davidoff Cancer Center, Rabin Medical Center, Petah- Tikva, Israel; 2 Faculty of Medical and Health Sciences, Tel Aviv University, Tel Aviv, Israel; 3 Department of Medicine A, Rabin Medical Center, Beilinson Campus, Petah-Tikva, Israel; 4 Division of Hematology and Bone Marrow Transplantation, Chaim Sheba Medical Center, Ramat-Gan, Israel; 5 Department of Hematology, Sourasky Medical Center, Tel Aviv, Israel; 6 Department of Hematology and Bone Marrow Transplantation, Rambam Health Care Campus, Haifa, Israel; 7 Faculty of Medicine, Technion- Israel Institute of Technology, Haifa, Israel; 8 Department of Hematology, Meir Medical Center, Kfar Saba, Israel; 9 Department of Hematology, Hadassah Medical Center, Faculty of Medicine, Hebrew University of Jerusalem, Jerusalem, Israel

**Keywords:** Glofitamab, Diffuse large B-Cell lymphoma (DLBCL), T-cell engager, Real-world data, Relapsed/refractory lymphoma, CAR-T failure

## Abstract

Glofitamab, a CD20-directed CD3 T-cell engager, was recently FDA-approved after demonstrating a 52% overall response rate (ORR) and a 39% complete response (CR) rate in heavily pretreated diffuse large B-cell lymphoma (DLBCL) patients. However, real-world data on its efficacy and safety remain limited. This study evaluated glofitamab’s performance in clinical practice. We conducted a retrospective multicenter study of adults with relapsed/refractory (R/R) DLBCL treated via a national compassionate use program. Patients received at least one dose of glofitamab after failing ≥ 2 prior therapies. Recruitment spanned September 2020–January 2023. Outcomes included ORR, CR (per Lugano criteria), progression-free survival (PFS), and overall survival (OS). Adverse events were classified per ASTCT 2019 criteria, and risk factors for PFS and OS were assessed via logistic regression. Thirty-five patients from six Israeli centers were included (median age: 67 years; 66% male). The median number of prior therapies was 5, with 43% being primary refractory and 91% post-CAR-T therapy. ORR was 34%, with 14% achieving CR. Median PFS and OS were 2 and 4 months, respectively. Treatment was prematurely discontinued in 86%, mainly due to disease progression (46%) and to infections in responding patients (17%). Male sex was a significant risk factor for poor PFS and OS. In this real-world cohort, glofitamab’s efficacy was lower than in clinical trials, likely due to a more heavily pretreated population. However, its manageable toxicity supports its potential role in r/r DLBCL treatment.

## Introduction

Diffuse large B cell lymphoma (DLBCL) is the most common form of non-Hodgkin lymphoma (NHL). With immunochemotherapy of R-CHOP (rituximab, cyclophosphamide, doxorubicin, vincristine, and prednisone), more than 60% of patients can be cured [1]. With the addition of polatuzumab to R-CHP, progressionfree survival (PFS) improved to 76.7% in comparison to 70% in a matched R-CHOP group [2]. In the relapse/ refractory setting, chimeric antigen receptor Tcell (CAR-T) products, axicabtagene ciloleucel (axi-cel) and lisocabtagene maraleucel (liso-cel), were approved as second line by FDA for early relapse i.e. within 12 months, due to superior results as compared to standard of care [3, 4].

Nevertheless, a proportion of patients will not receive CAR T-cell therapy due to rapidly progressive disease, failure in manufacturing, limited accessibility or comorbidities [1]. Patients with relapsed DLBCL who are ineligible for autologous stem cell transplantation (ASCT) or CAR-T therapy have limited treatment options. Second-line therapies for these patients include tafasitamab plus lenalidomide [5], or the combination of polatuzumab vedotin, bendamustine, and rituximab [6]. However, more than 40% of patients will relapse after secondline therapy, even in the CAR-T cell therapy era, and these patients have shown to have dismal prognosis [7, 8]. The introduction of the CD3/CD19 bispecific antibody blinatumomab, approved for the treatment of B-cell acute lymphoblastic leukemia (B-ALL) in 2015, demonstrated the potential of this class in the treatment of other lymphoid malignancies [9]. This led to the development of the four CD3/CD20 bispecific antibodies available nowadays- mosunetuzumab for follicular lymphoma (FL) [10], glofitamab for DLBCL [11], epcoritamab [12, 13], and the recently EMA approved odronextamab both for DLBCL and FL [14].

Glofitamab is a T-cell engaging bispecific antibody (Ab) with a novel 2:1 structure, harboring bivalency for CD20 and monovalency for CD3, leading to the recruitment and redirection of patients’ T cells to eliminate the malignant B cells. In the pivotal phase II study, glofitamab has shown promising results with overall response rate (ORR) of 52%, complete response (CR) rate of 39%, and a median progression free survival (PFS) of 4.9 months [11]. Real-world data is still lacking, especially in the more frail and heavily pre-treated population including patients after CAR-T failure. In the present study, we summarize the efficacy and safety of glofitamab of the Israeli lymphoma study group.

## Patients and methods

### Patient population

This retrospective analysis included all patients above the age of 18 years with a histologically confirmed diagnosis of relapsed or refractory (R/R) DLBCL or high-grade B cell lymphoma (HGBL), after failing at least two prior lines of therapy, treated through a national compassionate program for glofitamab. The study was conducted in six medical centers in Israel. Patients included in the analysis received at least one dose of glofitamab. The study period was between September 2020 and January 2023. Glofitamab was given in accordance with the pivotal study protocol.: i.e., patients received pretreatment with obinutuzumab to mitigate cytokine release syndrome, followed by glofitamab monotherapy for 12 cycles as follows: Cycle 1: D1 obinutuzumab 1000 mg, D8 glofitamab 2.5 mg with hospitalization for at least 24 h for surveillance, D15 glofitamab 10 mg. From cycle 2 day 1 glofitamab was given in a fixed dose of 30 mg every 3 weeks for a total of 12 cycles [11]. 

Pre-treatment clinical parameters, retrieved from the patient electronic medical records, included age, sex, date of diagnosis, histological sub-type including evidence of MYC BCL-2 translocations, international prognostic index (IPI), the Eastern Cooperative Oncology Group (ECOG) performance status at diagnosis and the presence of extra-nodal disease. This study was performed in line with the principles of the Declaration of Helsinki. Institutional Review Boards of the participating centers approved the study protocols. Informed consent was waived.

Positron emission tomography with computerized tomography (PET/CT) was done prior to the initiation of treatment, at pre-determined time points which included after cycle 2, at the end of planned therapy or at any time point with suspected progression at the discretion of the treating physician. Response to therapy was assessed according to Deauville score and Lugano criteria [15].

Outcomes included overall response rate (ORR) and complete remission (CR) rate, defined by the Lugano criteria and duration of response. Progression free survival (PFS) and overall survival (OS) which were defined from the day of obinutuzimab infusion until event (progression or death). Safety data included hematological and non-hematological adverse events (AE), classified according to the CTCAE criteria version 5.0 [16]. Cytokine release syndrome (CRS) and immune effector cells-associated neurotoxicity syndrome (ICANS) were defined by the ASTCT consensus 2019 [17].

### Statistical analysis

The analysis was performed using the IBM SPSS statistics 26 (IBM Corporation, Armonk, New York). Continuous data are expressed as mean and standard deviation or as median and interquartile range (IQR, 25–75 percentiles) as appropriate. Categorical data are reported as frequencies and percentages. We established risk factors for PFS by univariable analysis followed by multivariable analysis using logistic regression. Odds ratios (OR) with 95% confidence intervals (CI) were calculated. Hosmer-Lemeshow statistic was used for goodness of fit. The risk of PFS and OS during the follow-up were estimated from a Kaplan–Meier plot.

## Results

### Demographics and disease characteristics

A total of 35 consecutive patients treated in six Israeli centers were included. Median age at the time of initiation of glofitamab was 67 years (59–74) and 66% (*n* = 23) of the patients were males. The median number of previous therapies was 5 (IQR, 4–6) and 97% (*n* = 34) received at least 4 prior lines of therapy. 43% (*n* = 15) were primary refractory to first line therapy, 91% (*n* = 32) had previously failed CAR-T cell therapy and 23% (*n* = 8) had underwent autologous stem cell transplantation (ASCT) [Table 1].

**Table 1:** Patients’ demographics

Patients Characteristics	*N* = 35
**Sex** (N, %)	
Female	12, 34%
Male	23, 66%
**Age, years** (median, range)	
At diagnosis	66, 54–72
At glofitamab initiation	67, 59–74
**ECOG** (N, %)	
0–2	34, 97%
3–4	1, 3%
**Previous lines of therapy**	
Number of prior Tx lines (median, range)	5, 3–6
> 3 previous Tx lines (N, %)	34, 97%
Primary refractory (N, %)	15, 43%
Previous CAR-T cell therapy (N, %)	32, 91%
Previous ASCT (N, %)	8, 23%
**Follow up, months** (median, range)	34, 18–57

Abbreviations: ECOG- Eastern Cooperative Oncology Group, CAR-T- chimeric antigen receptor T cell, ASCT- autologous stem cell transplant

63% (*n* = 22) were diagnosed with DLBCL, 14% (*n* = 5) with HGBL, and 23% (*n* = 8) with transformed indolent lymphoma. Transformation originated from FL (3 patients), Chronic Lymphocytic Leukemia (2), Marginal Zone Lymphoma (2) and Waldenstrom Macroglobulinemia (1). Sixteen patients had Germinal Center B-cell like (GCB) phenotype by Hans criteria, 13 cases were non-GCB and the rest were undetermined. Most patients (74%) had stage 4 disease at glofitamab initiation, with 29% presenting with bulky disease (> 10 cm), and 20% had bone marrow involvement [Table 2].

**Table 2:** Disease characteristics

Clinical Characteristics at glofitamab initiation	*N* = 35
**Histology** (N, %)	
DLBCL NOS	22, 63%
HGBL	5, 14%
Transformed from low grade B cell lymphoma	8, 23%
**Ann Arbor Stage (N, %)**	
1	3, 9%
2	3, 9%
3	3, 9%
4	26, 74%
**Bulky disease** (N, %)	
Yes	10, 29%
No	25, 71%
**High LDH (N, %)**	
Yes	30, 86%
No	4, 11%
Data unavailable	1, 3%
**BM involvement (N, %)**	
Yes	7, 20%
No	28, 80%
>1 **Extra nodal site** (N, %)	
Yes	24, 69%
No	11, 31%

Abbreviations: BM- bone marrow, DLBCL- Diffuse Large B cell Lymphoma, HGBL- High Grade B cell Lymphoma

### Outcomes

#### Safety

Adverse events included CRS in 37% (*n* = 13), and ICANS in 6% (*n* = 2) of patients. All CRS events were grade 1–2, and ICANS events were grade 1. CRS rates decreased with dose escalation, with 14 events observed after the 2.5 mg dose, 10 events after the 10 mg dose, and 7 CRS events reported after administration of the full 30 mg dose. Tocilizumab was given to 14% (*n* = 5) of the patients, including 8.5% (*n* = 3) after the first 2.5 mg dose, and 6% (*n* = 2) of the patients after the 10 mg dose, with no subsequent administration of tocilizumab from third cycle and on. The two cases of ICANs were observed with concurrent CRS, one event was after a 2.5 mg dose, and the second event was after the administration of 10 mg. Grade 3–4 infections were reported in 23% (*n* = 8) of patients, including 1 case of COVID-19, 2 cases of febrile neutropenia, 4 cases of pneumonia, and 1 case of bacterial sepsis. Grade 5 infections were reported in 14% (*n* = 5) of patients, including 2 cases of COVID-19, 1 case of pneumonia and 2 cases of bacterial sepsis. Among the eight patients with grade 3–4 infections, 2 discontinued glofitamab due to the infections - one eventually died of progressive disease, and one is still alive at last follow up. Grade 3–4 cytopenias occurred in 14% (*n* = 5); 8.5% (*n* = 3) experiencing neutropenia, 3% (*n* = 1) thrombocytopenia, and 3% (*n* = 1) experienced both neutropenia and thrombocytopenia.

#### Efficacy

**Fig. 1:**
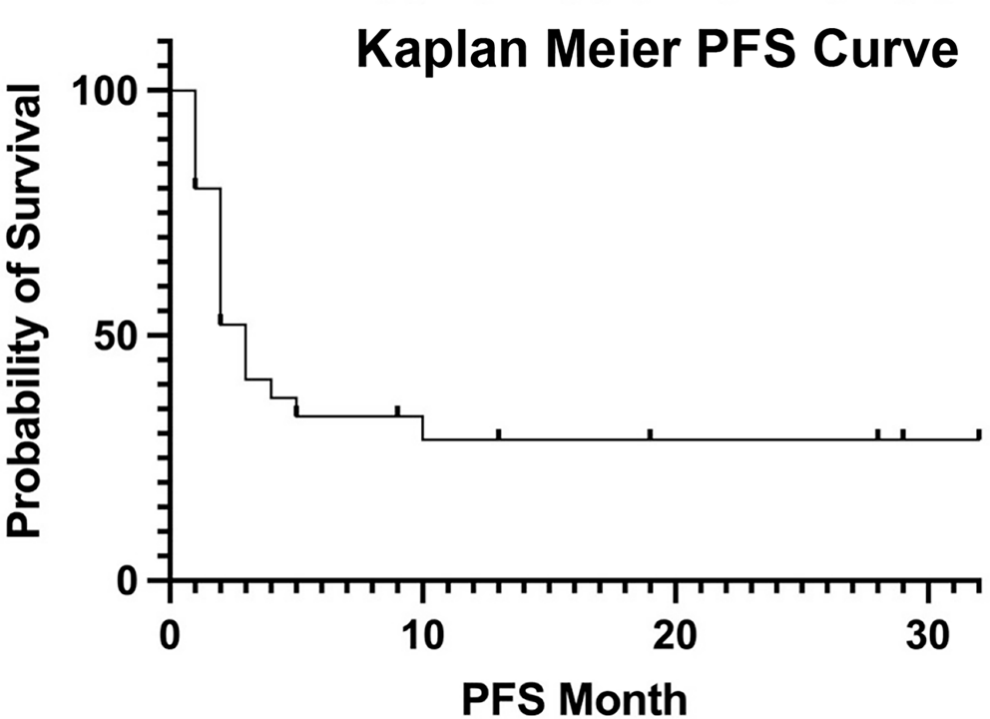
Progression free survival curve

**Fig. 2:**
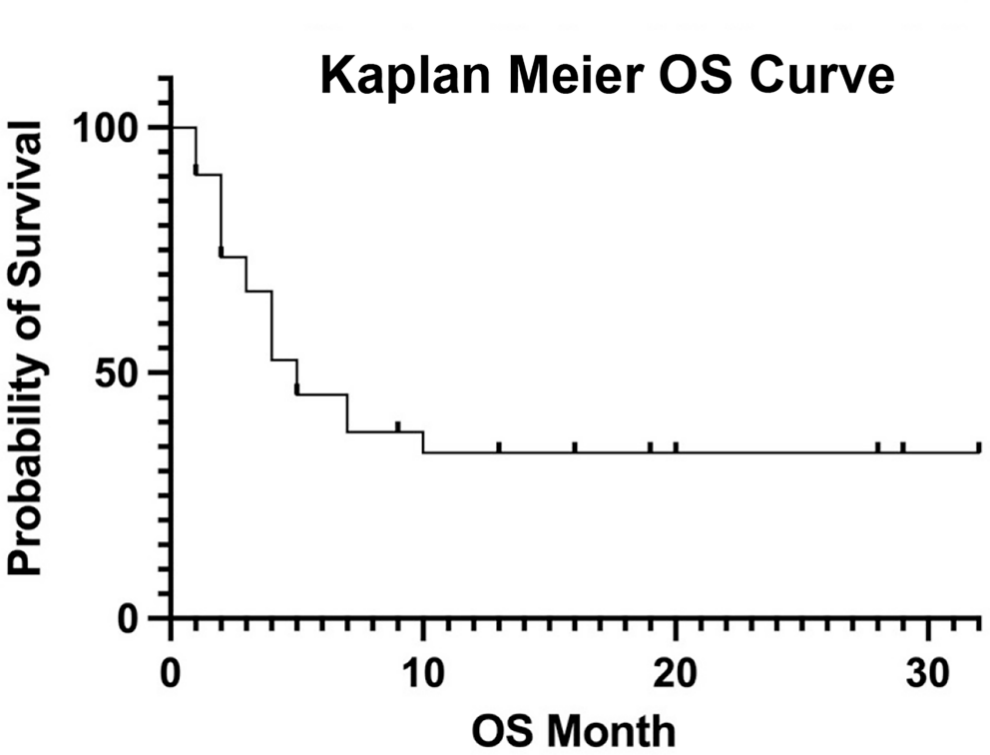
Overall survival curve

Median follow-up period was 34 months (IQR, 18–57). 34% achieved ORR with 14% achieving CR, as demonstrated by PET/CT performed after the second glofitamab cycle. Median PFS and OS were 2 (IQR, 1–3) and 4 months (IQR, 2–6), respectively [Figs. 1 and 2].

At the end of follow-up − 12 (34%) patients were alive, 21 died and 2 patients were lost to follow up. Causes of death included disease progression in 16 patients (76% of total causes of death) and grade 5 infections in 5 patients (24%). Treatment was discontinued earlier than 12 cycles in 86% of patients, mostly due to progressive disease (54%), and infections (14%). Two patients (6%) stopped treatment after 4 cycles and were then referred to allogeneic stem cell transplant. We performed uUnivariable analyses for identification of risk factors for PFS and OS using the following parameters before treatment: age, sex, bulky disease, LDH levels, thrombocytopenia, anemia, leukopenia, and for CRS after treatment. Only male sex was found to be a significant risk factor, and remained significant in the multivariant analysis (MVA) for PFS and OS (*p* = 0.02, *p* = 0.04 respectively) [Table 3].

**Table 3:** Multivariate for progression free survival and overall survival

Multivariable Analysis for Identification of Risk Factors After Glofitamab treatment				
variable	**OR for PFS (95% CI)**	**P value**	**OR for OS (95% CI)**	**P value**
Age	0.98 (0.8–1.1)	0.8	1 (0.9–1.1)	0.8
Male	0.03 (0.01–0.8)	0.04	0.06 (0.009-0.4))	0.007
Bulky disease	0.09 (0.003-2.5)	0.16	0.25 (0.025-2.4)	0.23
High LDH (at glofi initiation)	0.442 (0.05-40)	0.72	2.2 (0.5–8.5)	0.24
thrombocytopenia (at glofi initiation)	0.118 (0.003-4)	0.23	0.5 (0.05–5.8)	0.6
hypoalbuminemia	0 (0)	0.99	0.97 (0.14–6.3)	0.98
CRS	0.172 (0.008-3.7)	0.26	1 (0.9–1.1)	0.8

Abbreviations: CRS- cytokine release syndrome, OR- oddr ratio, CI- confidence interval, PFS- progression free survival, OS- overall survival

## Discussion

In this real world setting we retrospectively analyzed all patients treated with glofitamab in Israel between 2020 and 2023 as part of the compassionate use program. Our cohort included 35 patients, with a reported ORR of 34% and CR rate of 14%. Median PFS was only 2 months, with an OS of 4 months.

Glofitamab was approved by the FDA for R/R DLBCL based on the pivotal phase II study, demonstrating an ORR of 52%, CR of 39%, and a median PFS of 4.9 months [11]. The study recruited only patients with an ECOG of 0–1 and after at least two prior lines of treatment. Median prior lines of treatment was 3.

The observed efficacy outcomes are markedly inferior to what was reported in the pivotal study. One explanation for the inferior outcomes in our cohort might be the differences in study populations. Though our cohort had comparable demographics to the population of the pivotal trial in terms of age and sex, the population in our cohort was heavily pretreated. 97% of our patients received 4 or more lines of therapy before glofitamab, with a median of 5 prior lines compared to only 3 in the pivotal study. Another prominent difference between our cohort and the pivotal study cohort is the rate of prior exposure to CAR-T cell therapy—33% in the clinical trial (51 patients) vs. above 90% in our cohort—and prior ASCT—18% in the clinical trial vs. 23% in our cohort. In the pivotal study, 35% of the 51 patients with prior CAR-T exposure achieved CR, which is comparable to the 42% CR rate observed in the entire cohort (*n* = 155). Although there was no significant difference between patients with or without prior CAR-T exposure in the pivotal study analysis, the strength of this subgroup comparison may be limited. Moreover, almost all of the patients in our cohort had undergone prior immunotherapy, which might contribute to the inferior outcomes. Another contributing factor to the inferior results in our cohort might be a possible delay in treatment administration, as it was given within a compassionate use program.

Few real-world reports of glofitamab treatment for R/R DLBCL are available in the literature. A paper of 38 patients from Turkey reported an ORR of 37%, with a 21% CR rate. The median PFS was 3.3 months, and the median OS was 8.8 months. CRS developed in 27.9%, with 5 cases of grade ≥ 3 CRS, 7% had ICANS, and infections were reported in 20.9% of patients. In this report, ORR was comparable to our data, though the CR rate was higher, even if not as in the pivotal trial. Similar to our cohort, the median number of prior lines of therapy was higher than in the pivotal trial, with a median of 4 prior lines of treatment. This supports our explanation of the inferior outcomes in a real-world setting, where the population is often heavily pretreated, and disease is more progressed at the time of treatment. Nevertheless, none of the patients in this cohort had prior CAR-T cell exposure, as this treatment is not available in Turkey [18]. Another paper from Taiwan reported outcomes that were closer to those reported in the pivotal trial, with a 56% ORR and a 23% CR rate. The estimated PFS was 3.2 months, and the median OS was 8.4 months. 47% of patients had CRS after the first dose, 50% after the second dose and 12.5% after the third dose. 7% of reported CRS cases were grade 3 or more, no ICANs cases were reported, three patients discontinued therapy due to infections [19]. Of note, the cohort of the Taiwanese group, which reported better outcomes than we observed, also did not include patients post CAR-T therapy due to availability issues, whereas in our cohort most of the patients had prior exposure to CAR-T (91%). Again, though the pivotal trial’s sub-analysis did not demonstrate an effect of prior CAR-t exposure on outcomes, this should be evaluated in the real-world setting. As CAR-T became the standard of care for second line therapy for patients with refractory DLBCL or with early relapse [8], it is important to evaluate the effectiveness of bispecific antibodies in the post CAR-T relapses, since CAR-T therapy may have an effect on response to T cell engaging methods. The first report on glofitamab treatment in post CAR-T patients was published in 2022 with nine patients with progressive DLBCL after preceding CAR T-cell therapy. ORR was 67%, CR 44% (4 patients), and CRS was observed in two patients (grade 2 in both patients) [20]. In the 2023 ASH annual meeting, Sesques et al. presented the primary analysis of a phase II multicenter study using glofitamab in 63 pts with R/R non-Hodgkin B-cell lymphoma (DLBCL and non-DLBCL) after CAR T-cell therapy. Median age was 65 years, median number of prior therapies received was 3. CRS was observed in 14.3% all were grade 1–2, 28.6% experienced serious adverse events Gr ≥ 3, mainly infections. Treatment discontinuation occurred in 55.5%, mostly (44.4%) due to progression. With a median follow-up of 9.7 months, median PFS and OS were 4.9 and 17.6 months, respectively. Overall best metabolic response was 65.9% with complete metabolic response of (CMR) of 36.4%, and the median duration of CMR was 19.7 months. The response and survival rates were higher than previous reports, however, the complete data are yet to be published as this is an ongoing study [21]. Another recent report of BiTes treatment (glofitamab and epcoritamab) for R/R Large B cell lymphoma (LBCL) patients presented at the 2024 ASH annual meeting included 64 post CAR-T cell therapy patients. Among these patients, ORR was 54% with a CR of 33%. In a MVA of the data, only resistance to CAR-T therapy (patients not achieving CR/PR) was a significant predictor of lack of response to BiTes [22]. 

With emerging novel therapies for R/R LBCL, sequencing of therapies becomes one of the greatest interest questions. The durability of CAR-T in comparison to relatively short-term survival data with BiTes may favor treatment with CAR-T, especially in LBCL where the goal of treatment is curative. Nevertheless, data on the safety and efficacy of BITes for R/R LBCL are accumulating with longer term follow up periods. Furthermore, persistent antigen stimulation and tonic receptor signaling result in T-cell exhaustion and may compromise outcomes of sequenced T-cell–based immunotherapies [23]. Therefore, real-world data of sequenced immunotherapies outcomes to guide clinicians’ decisions are essential. Our data presents a retrospective cohort of R/R DLBCL patients treated with glofitamab after CAR-T exposure (*n* = 32), and the inferior outcome may suggest that the sequencing of treatments plays an important role in efficacy and should be taken into consideration when choosing treatment.

Adverse events (AEs) in our cohort included CRS in 37% of patients, in comparison to 64% in the pivotal trial, and 6% ICANS, in comparison to 8% in the pivotal trial. There were no grade 3–4 CRS/ICANS cases in our cohort, while 4% and 3% were reported in the pivotal trial, respectively. Grade 3–4 infections were reported in 37% of our patients, similar to the 38% reported in the pivotal trial. In our cohort, grade 5 infections occurred in 14% of patients (*n* = 5), compared to 1% (*n* = 2) reported in the pivotal trial. Several factors may explain this discrepancy. Our population was more heavily pretreated and included patients enrolled through a compassionate use program, often leading to delays in treatment initiation and further immunosuppression. Moreover, data were collected during the COVID-19 pandemic, further increasing the vulnerability of this patient population to infectious complications. These differences highlight the gap between outcomes observed in clinical trials and real-world experience. Furthermore, in our cohort 14% of patients stopped treatment due to AEs, while in the pivotal trial only 9% discontinued treatment. AEs in our cohort align closely with the data of the pivotal trial, and the CRS rate was even lower than in the pivotal study (37% vs. 63%) but similar to other real-world studies [18, 19]. This might be also due to overreporting in clinical trials. These results reassure the safety of glofitamab in R/R DLBCL and support its role in the treatment paradigm in this setting.

## Conclusion

Our data support the potential benefits of glofitamab in the heavily pre-treated population including patients with prior exposure to immune-based therapy, and suggest that incorporation of glofitamab in earlier lines of therapy might be more beneficial. The optimal sequence of immune-based therapy in heavily pre-treated lymphoma patients requires larger randomized trials.

## Data Availability

Data is provided within the manuscript.
